# Supplementary material for: Development of a deep learning based framework for classification of Indian venomous snakes integrated with explainable artificial intelligence for primary and emergency care providers
Source: PLoS Negl Trop Dis. 2026 Jun 5;20(6):e0014147. doi: 10.1371/journal.pntd.0014147 (PMC13271475; doi:10.1371/journal.pntd.0014147)
Supplement: Attachment — Submitted filename: VENOMS.AI Response to Reviewers.docx [file pntd.0014147.s002.docx]

**RESPONSE TO REVIEWERS**

| **Reviewer comment** | **Response** |
| --- | --- |
| **REVIEWER 1** | |
| 2nd paragraph introduction: “These include the Hump-nosed pit viper, Malabar pit viper, and King cobra, for which Indian polyvalent ASV provides little to no benefit.”  Provide scientific name for all species upon first mention. It is worth noting that the king cobra referred to here is Ophiophagus kaalinga. | Modifications made as advised |
| 2nd paragraph introduction: “Additionally, other significant species like the monocled cobra (Naja kaouthia) in northeastern India, various pit viper types, and regional variants such as Sochurek's saw scaled viper (Echis carinatus sochureki) are inadequately addressed by the existing polyvalent antivenoms. Please provide citations for this sentence. | Citations has been added as suggested. |
| 3rd paragraph introduction: “The binary distinction between venomous and non-venomous snakes is not adequately covered in the literature, even though this information is crucial for clinical triage.” Are you referring to medically important species? This isn’t really a “binary” distinction between venomous and non-venomous as many non-front-fanged snakes possess venoms but are not clinically important. Please clarify this sentence. | We would like to clarify that our current dataset is limited to 20 snake species and was primarily developed to test our hypothesis and build an initial AI-based classifier. We intend to expand this dataset in subsequent versions by incorporating additional species, enabling region-specific model training and improving.  Currently model is designed to classify snake species based on their venom status, irrespective of whether the venom is considered medically significant. However, for clinical application, we do not intend to rely solely on this simplified classification. Instead, future versions of the model will incorporate species-level and syndrome-based classification to better support clinical decision-making.  This approach is also justified by the fact that even species traditionally considered to be of low medical significance may, in certain circumstances, produce clinically relevant reactions, supporting the need for a more detailed classification approach |
| 2.2: “Bamboo Pit Viper, Beddome’s Keelback, Buff-striped Keelback, Cat Snake, Checkered Keelback, Common Krait, Common Wolf Snake, Green Vine Snake, Hump-nosed Pit Viper, Indian Rock Python, King Cobra, Malabar Pit Viper, Ornate Flying Snake, Rat Snake, Russell’s Kukri, Russell’s Viper, Saw-scaled Viper, Sea Krait, Spectacled Cobra, and Whitaker’s Boa, as shown in Fig 1.” Please include scientific names for all species. Note also that king cobras in India now comprise two species, Ophiophagus hannah in the north and O. kaalinga in the south. Several of these common names are apparently referring to entire genera (i.e. bamboo pit viper, cat snake, sea krait, etc), and simply listing for example Trimeresurus spp. may suffice here. | We agree that several of the common names used may refer to broader genera. However, in the context of our dataset, each label corresponds to specific species represented in the training images. Using genus-level terms (e.g., Trimeresurus spp.) could imply inclusion of additional species that were not part of the dataset.  We have therefore included the scientific names for each species in the revised manuscript.  With regard to king cobras, we note that our dataset includes only individuals from southern India, and therefore corresponds to *Ophiophagus kaalinga* |
| Figure 1 Caption. Please list the names and location (common and scientific) of the snakes in this figure. I suggest adding A, B, C, D to the photos to differentiate them.  Some context on the type of observation would also be useful (killed by resident, encountered in home, etc). | Caption has been revised as suggested |
| Figure 2 Caption: Please provide additional detail in this caption briefly explaining the process in each of the 4 steps shown in the figure. | The Figure 2 caption has been revised to briefly explain each step of the pipeline individually. |
| Figure 3 Caption: Please adjust the caption for punctuation and spacing. There should be commas separating each term. | The caption has been corrected to include commas separating each augmentation term, with consistent capitalization and spacing throughout. |
| Figure 5 Caption: Please adjust punctuation and spacing for this sentence. | The erroneous numerical characters and inconsistent spacing have been removed, and the caption has been reformatted with correct punctuation and uniform spacing throughout. |
| Table 1 Caption: Add a bit of information on which model performed best and maybe consider bolding this one in the table. | The caption has been updated to explicitly identify ResNeXt-50 (32×4d) as the best-performing model, and its corresponding row in Table 1 has been bolded to provide clear visual distinction from the remaining architectures. The revised caption reads:  *"Table 1: Comparative performance metrics of the four evaluated deep learning architectures. ResNeXt-50 (32×4d), highlighted in bold, outperformed all other models with the highest test accuracy (96.73%), sensitivity (93.46%), specificity (100%), and F1-score (96.73)."* |
| Figure 6 Caption: Please explain each plot individually via labelling these A and B, adjust the figure accordingly. | The figure has been replaced with panels (A) and (B), and the caption revised to describe each plot individually with their respective performance values and clinical relevance. |
| Figure 7 Caption: Please provide species identity and context (location, roadkill?) for this image. | Modification to captions done as advised. |
| Figure 8 Caption: Please provide species identity and context for this image. | Modification to captions done as advised. |
| 9th Paragraph Results: Python molurus should be italicized. | *Python molurus* has been italicized in the 9th paragraph of the Results section |
| Figure 9 Caption: This caption should be written out as a full sentence, please provide species identity and context for snake image. | The caption has been rewritten as complete sentences, with species identity (*Python molurus*) and clinical context — correct non-venomous classification at 96.53% confidence — explicitly stated for the snake image in panel (B). |
| 4.1 Study limitations: “Additionally, all images were sourced from a single institution, limiting claims of geographic and demographic generalizability.” This is an important point, as several of these medically important snakes in India have bewildering phenotypic variation that might complicate assignment of identity. It would be good to have add a sentence within addressing this with references. Naja naja (10.11646/ZOOTAXA.5346.4.3) and Naja kaouthia (<https://doi.org/10.11646/zootaxa.5717.4.2>) both have dramatic intraspecific variation that might complicate image assignment, and several of the groups that you’ve listed at genus level (i.e. Trimeresurus spp.) also have a good deal of variation (10.3897/vz.71.e66239). This is worth a little more written context and supporting references. | Modifications have been made as advised. |
| Data are generally well presented, but in several areas figure and table captions lack sufficient detail. I have clarified in individual comments where additional information is needed | Modifications have been made in figure and table captions as suggested. |
| This study used a dataset of images of nonvenomous and venomous snakes to train four AI species identification models. These models performed well to varying degrees, suggesting that models may be an effective way to determine snake identities in the case of envenoming occurring in remote areas or in the absence of individuals capable of identifying venomous snakes. This work holds promise for regions where high snakebite tolls are exacerbated by substandard medical care, and while they do not serve as a replacement for expert ID, they may provide a suitable substitute for emergency cases. The manuscript is well written, and the authors do a good job of explaining the process of their models and limitations associated with them. I congratulate the authors on a nicely done project that holds potential to facilitate improved snakebite management in India. | We thank the reviewer of their valuable feedback. |
| **REVIEWER 2** | |
| The study was detailed in its methods, aims were met and the study design appropriate. | - |
| The article is sufficient in detail, matches the aims and the figures are well constructed. | - |
| All of the above are clearly stated; however, there could be greater detail in the discussions as to how the machine learning approach can be further optimised with the addition of biological features distinctive of the snake species. For example in Australia, the mildly/non- venomous Colubridae have narrow ventral scales that project to roughly halfway of the side of the snake. Elapidae (the exclusively venomous snakes) have flat and wide ventral scales and the pythons have narrow and flat ventral scales. Domain specific knowledge may improve the model significantly. | We agree that incorporating domain-specific biological knowledge into model architecture is a promising direction for improving classification performance. We have revised the manuscript with how this learning approach can be further optimized. |
| Accept | - |
| The article is well composed and very innovative. It has beyond sufficient merit to be published. | - |
| **REVIEWER 3** | |
| No revisions | - |
| No revisions | - |
| No revisions | - |
| No revisions | - |
| Nicely crafted.  May be need of the hour. Well explained by authors, though it is a tool, Clinicians should approach a snake bite victim by syndromic approach. May be helpful to clinicians in odd hours: 12 midnight to early morning. In which time everybody are taking rest in bed. Machine is a machine.  A robust trial is needed. Black krait (Bungarus niger/Lividus) which are causing numerous fatalities in Northeast India closely resembles to Rats snake. In these scenarios, sometimes machine (AI) may fail completely to differentiate venomous black krait and nonvenomous Rats snake. | We thank reviewers for their feedback. Our intention is for this model to serve as a supportive adjunct, particularly in resource-constrained setting, where access to immediate expert consultation may be limited. We agree that this tool is not a substitute for clinical judgment, but rather an aid to assist clinicians in early recognition and decision-making. |
