# Supplementary material for: Development of a deep learning based framework for classification of Indian venomous snakes integrated with explainable artificial intelligence for primary and emergency care providers
Source: PLoS Negl Trop Dis. 2026 Jun 5;20(6):e0014147. doi: 10.1371/journal.pntd.0014147 (PMC13271475; doi:10.1371/journal.pntd.0014147)
Supplement: Attachment — Submitted filename: Response to reviewers.docx [file pntd.0014147.s003.docx]

| 3rd paragraph introduction: “The binary distinction between venomous and non-venomous snakes is not adequately covered in the literature, even though this information is crucial for clinical triage.”  Are you referring to medically important species? This isn’t really a “binary” distinction between venomous and non-venomous as many non-front-fanged snakes possess venoms but are not clinically important. Please clarify this sentence.  **This section has not been clarified. | This justification had been provided in the previous rebuttal.  “We would like to clarify that our current dataset is limited to 20 snake species and was primarily developed to test our hypothesis and build an initial AI-based classifier. We intend to expand this dataset in subsequent versions by incorporating additional species, enabling region-specific model training and improving.  Currently model is designed to classify snake species based on their venom status, irrespective of whether the venom is considered medically significant. However, for clinical application, we do not intend to rely solely on this simplified classification. Instead, future versions of the model will incorporate species-level and syndrome-based classification to better support clinical decision-making.  This approach is also justified by the fact that even species traditionally considered to be of low medical significance may, in certain circumstances, produce clinically relevant reactions, supporting the need for a more detailed classification approach.’’  We have now incorporated the same in the revised manuscript. |
| --- | --- |
| 2.2: “Bamboo Pit Viper, Beddome’s Keelback, Buff-striped Keelback, Cat Snake, Checkered Keelback, Common Krait, Common Wolf Snake, Green Vine Snake, Hump-nosed Pit Viper, Indian Rock Python, King Cobra, Malabar Pit Viper, Ornate Flying Snake, Rat Snake, Russell’s Kukri, Russell’s Viper, Saw-scaled Viper, Sea Krait, Spectacled Cobra, and Whitaker’s Boa, as shown in Fig 1.”  Please include scientific names for all species. Note also that king cobras in India now comprise two species, Ophiophagus hannah in the north and O. kaalinga in the south. Several of these common names are apparently referring to entire genera (i.e. bamboo pit viper, cat snake, sea krait, etc), and simply listing for example Trimeresurus spp. may suffice here.  **You refer to the green vine snake here at Oxybelis fulgidus – this is a South American species not present in India. I assume that the snake you mean to refer to is Ahaetulla spp. Please correct this. | We thank the reviewer for identifying this error and we apologize for the same. The scientific name has been corrected from *Oxybelis fulgidus* to *Ahaetulla spp.* |
| Figure 1 Caption. Please list the names and location (common and scientific) of the snakes in this figure. I suggest adding A, B, C, D to the photos to differentiate them. Some context on the type of observation would also be useful (killed by resident, encountered in home, etc).  **The genus name Lydocon needs to be written in italics. | Modifications have been made as advised. |
| 9th Paragraph Results: Python molurus should be italicized.  **This correction has been left unaddressed | The scientific name *Python molurus* has now been italicized as advised. |
| Figure 9 Caption: This caption should be written out as a full sentence, please provide species identity and context for snake image.  **This figure caption still does not read as a sentence, more explanation is needed about what is happening here. | The caption for Figure 9 has been revised into a complete descriptive sentence. It now explains the deployment workflow, including upload of a snake image into the trained model, automated prediction of venom status with confidence percentage output, and subsequent validation by a trained expert |
| 4.1 Study limitations: “Additionally, all images were sourced from a single institution, limiting claims of geographic and demographic generalizability.”  This is an important point, as several of these medically important snakes in India have bewildering phenotypic variation that might complicate assignment of identity. It would be good to have add a sentence within addressing this with references. Naja naja (10.11646/ZOOTAXA.5346.4.3) and Naja kaouthia (https://doi.org/10.11646/zootaxa.5717.4.2) both have dramatic intraspecific variation, and then several of the groups that you’ve listed at genus level (i.e. Trimeresurus spp. also have a good deal of variation; 10.3897/vz.71.e66239). This is worth a little bit more context.  **I appreciate that modifications have made here, but a couple of spelling errors remain: “particular;y”, “intrasepcifc”, “varions” , “demontstate”. Additionally, Craspedocephalus needs to be written in italics. | Modifications have been made as advised. |
