# Supplementary material for: Development of a deep learning based framework for classification of Indian venomous snakes integrated with explainable artificial intelligence for primary and emergency care providers
Source: PLoS Negl Trop Dis. 2026 Jun 5;20(6):e0014147. doi: 10.1371/journal.pntd.0014147 (PMC13271475; doi:10.1371/journal.pntd.0014147)
Supplement: Peer review history

# Peer review: Decision Letter 0

**DOI:** 10.1371/journal.pntd.0014147.r001

**Authors:** Wuelton Monteiro

6 Apr 2026

PNTD-D-26-00482

Development of a Deep Learning Based Framework for Classification of Indian Venomous Snakes Integrated with Explainable Artificial Intelligence for primary and emergency care providers

PLOS Neglected Tropical Diseases

Dear Dr. Sirur,

Thank you for submitting your manuscript to PLOS Neglected Tropical Diseases. After careful consideration, we feel that it has merit but does not fully meet PLOS Neglected Tropical Diseases's publication criteria as it currently stands. Therefore, we invite you to submit a revised version of the manuscript that addresses the points raised during the review process.

Please submit your revised manuscript by Jun 05 2026 11:59PM. If you will need more time than this to complete your revisions, please reply to this message or contact the journal office at plosntds@plos.org. When you're ready to submit your revision, log on to https://www.editorialmanager.com/pntd/ and select the 'Submissions Needing Revision' folder to locate your manuscript file.

Please include the following items when submitting your revised manuscript:

* A letter that responds to each point raised by the editor and reviewer(s). You should upload this letter as a separate file labeled 'Response to Reviewers'. This file does not need to include responses to any formatting updates and technical items listed in the 'Journal Requirements' section below.

* A marked-up copy of your manuscript that highlights changes made to the original version. You should upload this as a separate file labeled 'Revised Manuscript with Track Changes'.

* An unmarked version of your revised paper without tracked changes. You should upload this as a separate file labeled 'Manuscript'.

If you would like to make changes to your financial disclosure, competing interests statement, or data availability statement, please make these updates within the submission form at the time of resubmission. Guidelines for resubmitting your figure files are available below the reviewer comments at the end of this letter.

We look forward to receiving your revised manuscript.

Kind regards,

Wuelton Monteiro, Ph.D.

Section Editor

PLOS Neglected Tropical Diseases

Wuelton Monteiro

Section Editor

PLOS Neglected Tropical Diseases

Shaden Kamhawi

co-Editor-in-Chief

PLOS Neglected Tropical Diseases

orcid.org/0000-0003-4304-636XX

Paul Brindley

co-Editor-in-Chief

PLOS Neglected Tropical Diseases

orcid.org/0000-0003-1765-0002

**Journal Requirements:**

If the reviewer comments include a recommendation to cite specific previously published works, please review and evaluate these publications to determine whether they are relevant and should be cited. There is no requirement to cite these works unless the editor has indicated otherwise.

1) Please upload all main figures as separate Figure files in .tif or .eps format. For more information about how to convert and format your figure files please see our guidelines:

https://journals.plos.org/plosntds/s/figures

2) When completing the data availability statement of the submission form, you indicated that you will make your data available on acceptance. We strongly recommend all authors decide on a data sharing plan before acceptance, as the process can be lengthy and hold up publication timelines. Please note that, though access restrictions are acceptable now, your entire data will need to be made freely accessible if your manuscript is accepted for publication. This policy applies to all data except where public deposition would breach compliance with the protocol approved by your research ethics board. If you are unable to adhere to our open data policy, please kindly revise your statement to explain your reasoning and we will seek the editor's input on an exemption. Please be assured that, once you have provided your new statement, the assessment of your exemption will not hold up the peer review process.

3) Some material included in your submission may be copyrighted. According to PLOS's copyright policy, authors who use figures or other material (e.g., graphics, clipart, maps) from another author or copyright holder must demonstrate or obtain permission to publish this material under the Creative Commons Attribution 4.0 International (CC BY 4.0) License used by PLOS journals. Please closely review the details of PLOS's copyright requirements here: PLOS Licenses and Copyright. If you need to request permissions from a copyright holder, you may use the PLOS Content Copyright Permission Form Please respond directly to this email and provide any known details concerning your material's license terms and permissions required for reuse, even if you have not yet obtained copyright permissions or are unsure of your material's copyright compatibility. Once you have responded and addressed all other outstanding technical requirements, you may resubmit your manuscript within Editorial Manager. Potential Copyright Issues:

i) Please confirm (a) that you are the photographer of 1, 2, 3, 7, 8, and 9, or (b) provide written permission from the photographer to publish the photo(s) under our CC BY 4.0 license.

ii) The following Figure contains screenshots: 9A. We are not permitted to publish these under our CC-BY 4.0 license, websites are usually intellectual property and are copyrighted.This includes peripheral graphics of the web browser such as icons and button. We ask that you please remove or replace it.

**Reviewers' comments:**

Reviewer's Responses to Questions

**Key Review Criteria Required for Acceptance?**

As you describe the new analyses required for acceptance, please consider the following:

**Methods**

-Are the objectives of the study clearly articulated with a clear testable hypothesis stated?

-Is the study design appropriate to address the stated objectives?

-Is the population clearly described and appropriate for the hypothesis being tested?

-Is the sample size sufficient to ensure adequate power to address the hypothesis being tested?

-Were correct statistical analysis used to support conclusions?

-Are there concerns about ethical or regulatory requirements being met?

Reviewer #1: 2nd paragraph introduction: “These include the Hump-nosed pit viper, Malabar pit viper, and King cobra, for which Indian polyvalent ASV provides little to no benefit.”

Provide scientific name for all species upon first mention. It is worth noting that the king cobra referred to here is Ophiophagus kaalinga.

2nd paragraph introduction: “Additionally, other significant species like the monocled cobra (Naja kaouthia) in northeastern India, various pit viper types, and regional variants such as Sochurek's saw scaled viper (Echis carinatus sochureki) are inadequately addressed by the existing polyvalent antivenoms.

Please provide citations for this sentence.

3rd paragraph introduction: “The binary distinction between venomous and non-venomous snakes is not adequately covered in the literature, even though this information is crucial for clinical triage.”

Are you referring to medically important species? This isn’t really a “binary” distinction between venomous and non-venomous as many non-front-fanged snakes possess venoms but are not clinically important. Please clarify this sentence.

2.2: “Bamboo Pit Viper, Beddome’s Keelback, Buff-striped Keelback, Cat Snake, Checkered Keelback, Common Krait, Common Wolf Snake, Green Vine Snake, Hump-nosed Pit Viper, Indian Rock Python, King Cobra, Malabar Pit Viper, Ornate Flying Snake, Rat Snake, Russell’s Kukri, Russell’s Viper, Saw-scaled Viper, Sea Krait, Spectacled Cobra, and Whitaker’s Boa, as shown in Fig 1.”

Please include scientific names for all species. Note also that king cobras in India now comprise two species, Ophiophagus hannah in the north and O. kaalinga in the south. Several of these common names are apparently referring to entire genera (i.e. bamboo pit viper, cat snake, sea krait, etc), and simply listing for example Trimeresurus spp. may suffice here.

Figure 1 Caption. Please list the names and location (common and scientific) of the snakes in this figure. I suggest adding A, B, C, D to the photos to differentiate them. Some context on the type of observation would also be useful (killed by resident, encountered in home, etc).

Figure 2 Caption: Please provide additional detail in this caption briefly explaining the process in each of the 4 steps shown in the figure.

Figure 3 Caption: Please adjust the caption for punctuation and spacing. There should be commas separating each term.

Reviewer #2: The study was detailed in its methods, aims were met and the study design appropriate.

Reviewer #3: Yes

**********

**Results**

-Does the analysis presented match the analysis plan?

-Are the results clearly and completely presented?

-Are the figures (Tables, Images) of sufficient quality for clarity?

Reviewer #1: Figure 5 Caption: Please adjust punctuation and spacing for this sentence.

Table 1 Caption: Add a bit of information on which model performed best and maybe consider bolding this one in the table.

Figure 6 Caption: Please explain each plot individually via labelling these A and B, adjust the figure accordingly.

Figure 7 Caption: Please provide species identity and context (location, roadkill?) for this image.

Figure 8 Caption: Please provide species identity and context for this image.

9th Paragraph Results: Python molurus should be italicized.

Figure 9 Caption: This caption should be written out as a full sentence, please provide species identity and context for snake image.

Reviewer #2: The article is sufficient in detail, matches the aims and the figures are well constructed.

Reviewer #3: Yes

**********

**Conclusions**

-Are the conclusions supported by the data presented?

-Are the limitations of analysis clearly described?

-Do the authors discuss how these data can be helpful to advance our understanding of the topic under study?

-Is public health relevance addressed?

Reviewer #1: 4.1 Study limitations: “Additionally, all images were sourced from a single institution, limiting claims of geographic and demographic generalizability.”

This is an important point, as several of these medically important snakes in India have bewildering phenotypic variation that might complicate assignment of identity. It would be good to have add a sentence within addressing this with references. Naja naja (10.11646/ZOOTAXA.5346.4.3) and Naja kaouthia (https://doi.org/10.11646/zootaxa.5717.4.2) both have dramatic intraspecific variation that might complicate image assignment, and several of the groups that you’ve listed at genus level (i.e. Trimeresurus spp.) also have a good deal of variation (10.3897/vz.71.e66239). This is worth a little more written context and supporting references.

Reviewer #2: All of the above are clearly stated; however, there could be greater detail in the discussions as to how the machine learning approach can be further optimised with the addition of biological features distinctive of the snake species. For example in Australia, the mildly/non- venomous Colubridae have narrow ventral scales that project to roughly half way of the side of the snake. Elapidae (the exclusively venomous snakes) have flat and wide ventral scales and the pythons have narrow and flat ventral scales. Domain specific knowledge may improve the model significantly.

Reviewer #3: Yes

**********

**Editorial and Data Presentation Modifications?**

Use this section for editorial suggestions as well as relatively minor modifications of existing data that would enhance clarity. If the only modifications needed are minor and/or editorial, you may wish to recommend “Minor Revision” or “Accept”.

Reviewer #1: Data are generally well presented, but in several areas figure and table captions lack sufficient detail. I have clarified in individual comments where additional information is needed.

Reviewer #2: Accept.

Reviewer #3: (No Response)

**********

**Summary and General Comments**

Use this section to provide overall comments, discuss strengths/weaknesses of the study, novelty, significance, general execution and scholarship. You may also include additional comments for the author, including concerns about dual publication, research ethics, or publication ethics. If requesting major revision, please articulate the new experiments that are needed.

Reviewer #1: This study used a dataset of images of nonvenomous and venomous snakes to train four AI species identification models. These models performed well to varying degrees, suggesting that models may be an effective way to determine snake identities in the case of envenoming occurring in remote areas or in the absence of individuals capable of identifying venomous snakes. This work holds promise for regions where high snakebite tolls are exacerbated by substandard medical care, and while they do not serve as a replacement for expert ID, they may provide a suitable substitute for emergency cases. The manuscript is well written, and the authors do a good job of explaining the process of their models and limitations associated with them. I congratulate the authors on a nicely done project that holds potential to facilitate improved snakebite management in India.

Reviewer #2: The article is well composed and very innovative. It has beyond sufficient merit to be published.

Reviewer #3: Nicely crafted.

May be need of the hour.

Well explained by authors,though it is a tool,Clinicians should approach a snake bite victims by syndromic approach.

May be helpful to clinicians in odd hours : 12 midnight to early morning.In which time everybody are taking rest in bed.

Machine is a machine.A robust trial is needed.Black krait(Bungarus niger/Lividus) which are causing numerous fatalities in North East India closely resembles to Rats snake.In these scenarios, sometimes machine(AI) may fail completely to differentiate venomous black krait and nonvenomous Rats snake.

**********

PLOS authors have the option to publish the peer review history of their article (what does this mean?). If published, this will include your full peer review and any attached files.

If you choose “no”, your identity will remain anonymous but your review may still be made public.

**Do you want your identity to be public for this peer review?**  For information about this choice, including consent withdrawal, please see our Privacy Policy.

Reviewer #1: **Yes:** Neil Balchan

Reviewer #2: **Yes:** Thomas Stocker

Reviewer #3: **Yes:** Dr Surajit Giri

**Figure resubmission:**

While revising your submission, we strongly recommend that you use PLOS’s NAAS tool (https://ngplosjournals.pagemajik.ai/artanalysis) to test your figure files. NAAS can convert your figure files to the TIFF file type and meet basic requirements (such as print size, resolution), or provide you with a report on issues that do not meet our requirements and that NAAS cannot fix.

After uploading your figures to PLOS’s NAAS tool - https://ngplosjournals.pagemajik.ai/artanalysis, NAAS will process the files provided and display the results in the "Uploaded Files" section of the page as the processing is complete. If the uploaded figures meet our requirements (or NAAS is able to fix the files to meet our requirements), the figure will be marked as "fixed" above. If NAAS is unable to fix the files, a red "failed" label will appear above. When NAAS has confirmed that the figure files meet our requirements, please download the file via the download option, and include these NAAS processed figure files when submitting your revised manuscript.

**Reproducibility:**

To enhance the reproducibility of your results, we recommend that authors of applicable studies deposit laboratory protocols in protocols.io, where a protocol can be assigned its own identifier (DOI) such that it can be cited independently in the future. Additionally, PLOS ONE offers an option to publish peer-reviewed clinical study protocols. Read more information on sharing protocols at https://plos.org/protocols?utm_medium=editorial-email&utm_source=authorletters&utm_campaign=protocols

# Author comment: Author response to Decision Letter 1

**DOI:** 10.1371/journal.pntd.0014147.r002

21 Apr 2026

Attachment Submitted filename: VENOMS.AI Response to Reviewers.docx

# Peer review: Decision Letter 1

**DOI:** 10.1371/journal.pntd.0014147.r003

**Authors:** Wuelton Monteiro

8 May 2026

PNTD-D-26-00482R1

Development of a Deep Learning Based Framework for Classification of Indian Venomous Snakes Integrated with Explainable Artificial Intelligence for primary and emergency care providers

PLOS Neglected Tropical Diseases

Dear Dr. Sirur,

Thank you for submitting your manuscript to PLOS Neglected Tropical Diseases. After careful consideration, we feel that it has merit but does not fully meet PLOS Neglected Tropical Diseases's publication criteria as it currently stands. Therefore, we invite you to submit a revised version of the manuscript that addresses the points raised during the review process.

Please submit your revised manuscript by Jun 07 2026 11:59PM. If you will need more time than this to complete your revisions, please reply to this message or contact the journal office at plosntds@plos.org. When you're ready to submit your revision, log on to https://www.editorialmanager.com/pntd/ and select the 'Submissions Needing Revision' folder to locate your manuscript file.

Please include the following items when submitting your revised manuscript:

* A letter that responds to each point raised by the editor and reviewer(s). You should upload this letter as a separate file labeled 'Response to Reviewers'. This file does not need to include responses to any formatting updates and technical items listed in the 'Journal Requirements' section below.

* A marked-up copy of your manuscript that highlights changes made to the original version. You should upload this as a separate file labeled 'Revised Manuscript with Track Changes'.

* An unmarked version of your revised paper without tracked changes. You should upload this as a separate file labeled 'Manuscript'.

If you would like to make changes to your financial disclosure, competing interests statement, or data availability statement, please make these updates within the submission form at the time of resubmission. Guidelines for resubmitting your figure files are available below the reviewer comments at the end of this letter.

As the corresponding author, your ORCID iD is verified in the submission system and will appear in the published article. PLOS supports the use of ORCID, and we encourage all coauthors to register for an ORCID iD and use it as well. Please encourage your coauthors to verify their ORCID iD within the submission system before final acceptance, as unverified ORCID iDs will not appear in the published article. Only the individual author can complete the verification step; PLOS staff cannot verify ORCID iDs on behalf of authors.

We look forward to receiving your revised manuscript.

Kind regards,

Wuelton Monteiro, Ph.D.

Section Editor

PLOS Neglected Tropical Diseases

Wuelton Monteiro

Section Editor

PLOS Neglected Tropical Diseases

Shaden Kamhawi

co-Editor-in-Chief

PLOS Neglected Tropical Diseases

orcid.org/0000-0003-4304-636XX

Paul Brindley

co-Editor-in-Chief

PLOS Neglected Tropical Diseases

orcid.org/0000-0003-1765-0002

**Journal Requirements:**

1) When completing the data availability statement of the submission form, you indicated that you will make your data available on acceptance. We strongly recommend all authors decide on a data sharing plan before acceptance, as the process can be lengthy and hold up publication timelines. Please note that, though access restrictions are acceptable now, your entire data will need to be made freely accessible if your manuscript is accepted for publication. This policy applies to all data except where public deposition would breach compliance with the protocol approved by your research ethics board. If you are unable to adhere to our open data policy, please kindly revise your statement to explain your reasoning and we will seek the editor's input on an exemption. Please be assured that, once you have provided your new statement, the assessment of your exemption will not hold up the peer review process.

Note: If the reviewer comments include a recommendation to cite specific previously published works, please review and evaluate these publications to determine whether they are relevant and should be cited. There is no requirement to cite these works unless the editor has indicated otherwise.

**Reviewers' comments:**

Reviewer's Responses to Questions

**Key Review Criteria Required for Acceptance?**

As you describe the new analyses required for acceptance, please consider the following:

**Methods**

-Are the objectives of the study clearly articulated with a clear testable hypothesis stated?

-Is the study design appropriate to address the stated objectives?

-Is the population clearly described and appropriate for the hypothesis being tested?

-Is the sample size sufficient to ensure adequate power to address the hypothesis being tested?

-Were correct statistical analysis used to support conclusions?

-Are there concerns about ethical or regulatory requirements being met?

Reviewer #1: See provided comments.

**********

**Results**

-Does the analysis presented match the analysis plan?

-Are the results clearly and completely presented?

-Are the figures (Tables, Images) of sufficient quality for clarity?

Reviewer #1: See provided comments.

**********

**Conclusions**

-Are the conclusions supported by the data presented?

-Are the limitations of analysis clearly described?

-Do the authors discuss how these data can be helpful to advance our understanding of the topic under study?

-Is public health relevance addressed?

Reviewer #1: See provided comments.

**********

**Editorial and Data Presentation Modifications?**

Use this section for editorial suggestions as well as relatively minor modifications of existing data that would enhance clarity. If the only modifications needed are minor and/or editorial, you may wish to recommend “Minor Revision” or “Accept”.

Reviewer #1: Minor revisions

**********

**Summary and General Comments**

Use this section to provide overall comments, discuss strengths/weaknesses of the study, novelty, significance, general execution and scholarship. You may also include additional comments for the author, including concerns about dual publication, research ethics, or publication ethics. If requesting major revision, please articulate the new experiments that are needed.

Reviewer #1: The authors have addressed many of my comments from the first round of review, but several errors still need correction. My comments from prior that require additional edits are included below as noted.

Minor Comments

3rd paragraph introduction: “The binary distinction between venomous and non-venomous snakes is not adequately covered in the literature, even though this information is crucial for clinical triage.”

Are you referring to medically important species? This isn’t really a “binary” distinction between venomous and non-venomous as many non-front-fanged snakes possess venoms but are not clinically important. Please clarify this sentence.

**This section has not been clarified.

2.2: “Bamboo Pit Viper, Beddome’s Keelback, Buff-striped Keelback, Cat Snake, Checkered Keelback, Common Krait, Common Wolf Snake, Green Vine Snake, Hump-nosed Pit Viper, Indian Rock Python, King Cobra, Malabar Pit Viper, Ornate Flying Snake, Rat Snake, Russell’s Kukri, Russell’s Viper, Saw-scaled Viper, Sea Krait, Spectacled Cobra, and Whitaker’s Boa, as shown in Fig 1.”

Please include scientific names for all species. Note also that king cobras in India now comprise two species, Ophiophagus hannah in the north and O. kaalinga in the south. Several of these common names are apparently referring to entire genera (i.e. bamboo pit viper, cat snake, sea krait, etc), and simply listing for example Trimeresurus spp. may suffice here.

**You refer to the green vine snake here at Oxybelis fulgidus – this is a South American species not present in India. I assume that the snake you mean to refer to is Ahaetulla spp. Please correct this.

Figure 1 Caption. Please list the names and location (common and scientific) of the snakes in this figure. I suggest adding A, B, C, D to the photos to differentiate them. Some context on the type of observation would also be useful (killed by resident, encountered in home, etc).

**The genus name Lydocon needs to be written in italics.

9th Paragraph Results: Python molurus should be italicized.

**This correction has been left unaddressed.

Figure 9 Caption: This caption should be written out as a full sentence, please provide species identity and context for snake image.

**This figure caption still does not read as a sentence, more explanation is needed about what is happening here.

4.1 Study limitations: “Additionally, all images were sourced from a single institution, limiting claims of geographic and demographic generalizability.”

This is an important point, as several of these medically important snakes in India have bewildering phenotypic variation that might complicate assignment of identity. It would be good to have add a sentence within addressing this with references. Naja naja (10.11646/ZOOTAXA.5346.4.3) and Naja kaouthia (https://doi.org/10.11646/zootaxa.5717.4.2) both have dramatic intraspecific variation, and then several of the groups that you’ve listed at genus level (i.e. Trimeresurus spp. also have a good deal of variation; 10.3897/vz.71.e66239). This is worth a little bit more context.

**I appreciate that modifications have made here, but a couple of spelling errors remain: “particular;y”, “intrasepcifc”, “varions” , “demontstate”. Additionally, Craspedocephalus needs to be written in italics.

**********

PLOS authors have the option to publish the peer review history of their article (what does this mean?). If published, this will include your full peer review and any attached files.

If you choose “no”, your identity will remain anonymous but your review may still be made public.

**Do you want your identity to be public for this peer review?**  For information about this choice, including consent withdrawal, please see our Privacy Policy.

Reviewer #1: No

[NOTE: If reviewer comments were submitted as an attachment file, they will be attached to this email and accessible via the submission site. Please log into your account, locate the manuscript record, and check for the action link "View Attachments". If this link does not appear, there are no attachment files.]

**Figure resubmission:**

While revising your submission, we strongly recommend that you use PLOS’s NAAS tool (https://ngplosjournals.pagemajik.ai/artanalysis) to test your figure files. NAAS can convert your figure files to the TIFF file type and meet basic requirements (such as print size, resolution), or provide you with a report on issues that do not meet our requirements and that NAAS cannot fix.

After uploading your figures to PLOS’s NAAS tool - https://ngplosjournals.pagemajik.ai/artanalysis, NAAS will process the files provided and display the results in the "Uploaded Files" section of the page as the processing is complete. If the uploaded figures meet our requirements (or NAAS is able to fix the files to meet our requirements), the figure will be marked as "fixed" above. If NAAS is unable to fix the files, a red "failed" label will appear above. When NAAS has confirmed that the figure files meet our requirements, please download the file via the download option, and include these NAAS processed figure files when submitting your revised manuscript.

**Reproducibility:**

To enhance the reproducibility of your results, we recommend that authors of applicable studies deposit laboratory protocols in protocols.io, where a protocol can be assigned its own identifier (DOI) such that it can be cited independently in the future. Additionally, PLOS ONE offers an option to publish peer-reviewed clinical study protocols. Read more information on sharing protocols at https://plos.org/protocols?utm_medium=editorial-email&utm_source=authorletters&utm_campaign=protocols

# Author comment: Author response to Decision Letter 2

**DOI:** 10.1371/journal.pntd.0014147.r004

19 May 2026

Attachment Submitted filename: Response to reviewers.docx

# Editor report: Decision Letter 2

**DOI:** 10.1371/journal.pntd.0014147.r005

**Authors:** Wuelton Monteiro

20 May 2026

Dear Dr Sirur,

We are pleased to inform you that your manuscript 'Development of a Deep Learning Based Framework for Classification of Indian Venomous Snakes Integrated with Explainable Artificial Intelligence for primary and emergency care providers' has been provisionally accepted for publication in PLOS Neglected Tropical Diseases.

Before your manuscript can be formally accepted you will need to complete some formatting changes, which you will receive in a follow up email. A member of our team will be in touch with a set of requests.

Please note that your manuscript will not be scheduled for publication until you have made the required changes, so a swift response is appreciated.

IMPORTANT: The editorial review process is now complete. PLOS will only permit corrections to spelling, formatting or significant scientific errors from this point onwards. Requests for major changes, or any which affect the scientific understanding of your work, will cause delays to the publication date of your manuscript.

Should you, your institution's press office or the journal office choose to press release your paper, you will automatically be opted out of early publication. We ask that you notify us now if you or your institution is planning to press release the article. All press must be co-ordinated with PLOS.

Thank you again for supporting Open Access publishing; we are looking forward to publishing your work in PLOS Neglected Tropical Diseases.

Best regards,

Wuelton Monteiro, Ph.D.

Section Editor

PLOS Neglected Tropical Diseases

Wuelton Monteiro

Section Editor

PLOS Neglected Tropical Diseases

Shaden Kamhawi

co-Editor-in-Chief

PLOS Neglected Tropical Diseases

orcid.org/0000-0003-4304-636XX

Paul Brindley

co-Editor-in-Chief

PLOS Neglected Tropical Diseases

orcid.org/0000-0003-1765-0002

***********************************************************

p.p1 {margin: 0.0px 0.0px 0.0px 0.0px; line-height: 16.0px; font: 14.0px Arial; color: #323333; -webkit-text-stroke: #323333}span.s1 {font-kerning: none

# Editor report: Acceptance letter

**DOI:** 10.1371/journal.pntd.0014147.r006

**Authors:** Wuelton Monteiro

Dear Dr Sirur,

We are delighted to inform you that your manuscript, "Development of a Deep Learning Based Framework for Classification of Indian Venomous Snakes Integrated with Explainable Artificial Intelligence for primary and emergency care providers," has been formally accepted for publication in PLOS Neglected Tropical Diseases.

We have now passed your article onto the PLOS Production Department who will complete the rest of the publication process. All authors will receive a confirmation email upon publication.

The corresponding author will soon be receiving a typeset proof for review, to ensure errors have not been introduced during production. Please review the PDF proof of your manuscript carefully, as this is the last chance to correct any scientific or type-setting errors. Please note that major changes, or those which affect the scientific understanding of the work, will likely cause delays to the publication date of your manuscript. Note: Proofs for Front Matter articles (Editorial, Viewpoint, Symposium, Review, etc...) are generated on a different schedule and may not be made available as quickly.

Soon after your final files are uploaded, the early version of your manuscript will be published online unless you opted out of this process. The date of the early version will be your article's publication date. The final article will be published to the same URL, and all versions of the paper will be accessible to readers.

For Research Articles, you will receive an invoice from PLOS for your publication fee after your manuscript has reached the completed accept phase. If you receive an email requesting payment before acceptance or for any other service, this may be a phishing scheme. Learn how to identify phishing emails and protect your accounts at https://explore.plos.org/phishing.

Thank you again for supporting open-access publishing; we are looking forward to publishing your work in PLOS Neglected Tropical Diseases.

Best regards,

Shaden Kamhawi

co-Editor-in-Chief

PLOS Neglected Tropical Diseases

Paul Brindley

co-Editor-in-Chief

PLOS Neglected Tropical Diseases
